# The Effect of Smartphone Interventions on Patients With Chronic Obstructive Pulmonary Disease Exacerbations: A Systematic Review and Meta-Analysis

**DOI:** 10.2196/mhealth.5921

**Published:** 2016-09-01

**Authors:** Meshari Alwashmi, John Hawboldt, Erin Davis, Carlo Marra, John-Michael Gamble, Waseem Abu Ashour

**Affiliations:** ^1^ Memorial University School of Pharmacy St. John's, NL Canada

**Keywords:** pulmonary disease, chronic obstructive, telemedicine, smartphone, self care, disease progression, review, meta-analysis

## Abstract

**Background:**

The prevalence and mortality rates of chronic obstructive pulmonary disease (COPD) are increasing worldwide. Therefore, COPD remains a major public health problem. There is a growing interest in the use of smartphone technology for health promotion and disease management interventions. However, the effectiveness of smartphones in reducing the number of patients having a COPD exacerbation is poorly understood.

**Objective:**

To summarize and quantify the association between smartphone interventions and COPD exacerbations through a comprehensive systematic review and meta-analysis.

**Methods:**

A comprehensive search strategy was conducted across relevant databases (PubMed, Embase, Cochrane, CINHA, PsycINFO, and the Cochrane Library Medline) from inception to October 2015. We included studies that assessed the use of smartphone interventions in the reduction of COPD exacerbations compared with usual care. Full-text studies were excluded if the investigators did not use a smartphone device or did not report on COPD exacerbations. Observational studies, abstracts, and reviews were also excluded. Two reviewers extracted the data and conducted a risk of bias assessment using the US Preventive Services Task Force quality rating criteria. A random effects model was used to meta-analyze the results from included studies. Pooled odds ratios were used to measure the effectiveness of smartphone interventions on COPD exacerbations. Heterogeneity was measured using the *I*^2^statistic.

**Results:**

Of the 245 unique citations screened, 6 studies were included in the qualitative synthesis. Studies were relatively small with less than 100 participants in each study (range 30 to 99) and follow-up ranged from 4-9 months. The mean age was 70.5 years (SD 5.6) and 74% (281/380) were male. The studies varied in terms of country, type of smartphone intervention, frequency of data collection from the participants, and the feedback strategy. Three studies were included in the meta-analysis. The overall assessment of potential bias of the studies that were included in the meta-analysis was “Good” for one study and “Fair” for 2 studies. The pooled random effects odds ratio of patients having an exacerbation was 0.20 in patients using a smartphone intervention (95% CI 0.07-0.62), a reduction of 80% for smartphone interventions compared with usual care. However, there was moderate heterogeneity across the included studies (*I*^2^=59%).

**Conclusion:**

Although current literature on the role of smartphones in reducing COPD exacerbations is limited, findings from our review suggest that smartphones are useful in reducing the number of patients having a COPD exacerbation. Nevertheless, using smartphones require synergistic strategies to achieve the desired outcome. These results should be interpreted with caution due to the heterogeneity among the studies. Researchers should focus on conducting rigorous studies with adequately powered sample sizes to determine the validity and clinical utility of smartphone interventions in the management of COPD.

## Introduction

Chronic obstructive pulmonary disease (COPD) refers to a group of lung diseases that includes chronic bronchitis and emphysema. Often, the occurrence of COPD is associated with smoking [1]. The Global initiative for chronic Obstructive Lung Disease (GOLD) defines COPD as follows:

Chronic obstructive pulmonary disease (COPD), a common preventable and treatable disease, is characterized by persistent airflow limitation that is usually progressive and associated with an enhanced chronic inflammatory response in the airways and the lung to noxious particles or gases. Exacerbations and comorbidities contribute to the overall severity in individual patients [1]

The prevalence and mortality rates of COPD are increasing worldwide. Therefore, COPD remains a major public health problem. One of the major effects of COPD is a reduced physical activity level in the affected patients [2]. Although COPD is a preventable and treatable condition, it is the fourth leading cause of death in Canada [3].

GOLD defines a COPD exacerbation as an acute event characterized by a worsening of the patient’s respiratory symptoms that is beyond normal day-to-day variations and leads to a change in medication [1]. An acute exacerbation of COPD has detrimental effects on lung function, health-related quality of life, and exercise capacity [4]. According to the Canadian Institute for Health Information, COPD now accounts for the highest rate of hospital admission and readmission among major chronic illnesses in Canada [5]. The Conference Board of Canada has stated that the combined direct and indirect costs of COPD will increase from just under $4 billion in 2010 to roughly $9.5 billion by 2030, an increase of 140% [6]. Dynamic modeling has shown that any intervention that can reduce the number of exacerbations in a population will have a substantial impact on morbidity and costs of COPD [6,7].

Current advances in smartphones have allowed for opportunities to provide effective health promotion and disease management interventions. Several published studies indicate that smartphones can deliver effective interventions among various age groups and diseases [8-11]. Moreover, interventions delivered via a smartphone may empower patients to play a more active role in managing their health [9].

Recent improvements in smartphones suggest a potential for integration into COPD management. Effective COPD management could delay disease progression, reduce acute exacerbations, and improve quality of life [12]. Wang et al stated that a mobile phone–based system could provide an efficient home endurance exercise training program to improve exercise capacity, strengthen limb muscles and decrease systemic inflammation in COPD patients [13]. Another study indicated that smartphone-based collection of COPD symptom diaries allows patients to identify exacerbation symptoms at an early stage allowing for the opportunity for early intervention [14,15].

A thorough review of the literature is necessary to understand the gaps and challenges in the current use of smartphones in COPD management. It will inform the design of future smartphone apps that aim to limit COPD exacerbations. Therefore, we conducted a systematic review and meta-analysis to answer the following question:

In patients diagnosed with COPD, will using smart phone interventions, compared with not using smart phone interventions, reduce the number of patients that have at least one exacerbation?

## Methods

### Eligibility Criteria

We included randomized controlled trials and quasi-randomized studies that used smartphone interventions in patients with COPD. A smartphone was defined as a mobile phone that performs many of the functions of a computer, typically having a touchscreen interface, Internet access, and an operating system capable of running downloaded applications. Some smartphone interventions can also include the use of medical devices that transfer data to the smartphone or a Web-based platform for monitoring and analysis. Studies define COPD exacerbations differently due to the lack of a universally accepted objective definition of a COPD exacerbation. Some investigators define COPD based on drug use, reported symptoms, or emergency admission. As a result, we based our definition of exacerbation according to the GOLD criteria:

COPD exacerbation is an acute event characterized by a worsening of the patient’s respiratory symptoms that is beyond normal day-to-day variations and leads to a change in medication [1].

Studies that included additional medical conditions as well as COPD were retained if the outcomes specific to the COPD group were reported separately. All English and non-English language studies identified during the search were considered. Non-English language studies included an English abstract. The abstract was sufficient to apply the eligibility criteria. Observational studies, abstracts, and reviews were excluded. Studies without a control group were also excluded. Smartphones are carried everywhere, have constant Internet connections, and are used as communication devices. Therefore, studies that used only a tablet or Web-based intervention and not specifically a smartphone intervention were excluded.

### Search Strategy

A comprehensive literature search was conducted in consultation with a librarian with experience in conducting systematic reviews. The literature search was run from the inception of each database until October 14, 2015 using the methods recommended by the Preferred Reporting Items for Systematic Reviews and Meta-Analyses (PRISMA) guidelines [16]. Five electronic databases: PubMed, Embase, Cochrane, CINHA, PsycINFO, and the Cochrane Library were searched for published article that studied the effect of smartphone interventions on COPD exacerbations. The references of all included studies were examined for relevant articles. The researchers used key search terms to identify potential studies (see Table 1).

**Table 1:** Search terms for systematic review.

Search lines	Search terms
Line 1	(((((((((“obstructive lung disease”[Title/Abstract]) OR copd[Title/Abstract]) OR coad[Title/Abstract]) OR “chronic obstructive pulmonary disease”[Title/Abstract]) OR “chronic obstructive lung disease”[Title/Abstract]) OR “chronic obstructive airway* disease”[Title/Abstract])) OR (((((“Lung Diseases, Obstructive”[Mesh]) OR “Pulmonary Disease, Chronic Obstructive”[Mesh]) OR “COPD, Severe Early-Onset”[Supplementary Concept]) OR “Pulmonary Emphysema”[Mesh]) OR “Bronchitis, Chronic”[Mesh])))
2. AND	(((((((((((((((((((((((((((((“mobile phone”[Title/Abstract]) OR “smart phone”[Title/Abstract]) OR smartphone[Title/Abstract]) OR “cell phone”[Title/Abstract]) OR “personal digital assistant”[Title/Abstract]) OR PDA[Title/Abstract]) OR microcomputer[Title/Abstract]) OR blackberry[Title/Abstract]) OR nokia[Title/Abstract]) OR samsung[Title/Abstract]) OR “i phone”[Title/Abstract]) OR iphone[Title/Abstract]) OR symbian[Title/Abstract]) OR windows[Title/Abstract]) OR INQ[Title/Abstract]) OR ipad[Title/Abstract]) OR “i pad”[Title/Abstract]) OR ipod[Title/Abstract]) OR “i pod”[Title/Abstract]) OR mhealth[Title/Abstract]) OR “mobile health”[Title/Abstract]) OR “m health”[Title/Abstract]) OR “m-health”[Title/Abstract]) OR app[Title/Abstract]) OR HTC[Title/Abstract]) OR samsung[Title/Abstract]) OR apps[Title/Abstract])) OR ((((“Cell Phones”[Mesh]) OR “Computers, Handheld”[Mesh]) OR “Text Messaging”[Mesh]) OR “Telemedicine”[Mesh]))))
3. AND	((“Disease Progression”[Mesh]) OR exacerbation[Title/Abstract])

### Study Screening

Two authors (MA and WA) screened titles and abstracts for each unique citation. The screening process included removing duplicates and excluding studies that were not related to COPD or telemonitoring. The remaining full-text studies were then assessed for eligibility. Full-text studies were excluded if the investigators did not use a smartphone device or did not report on COPD exacerbations. The reviewers also included studies that reported the rate of COPD exacerbations in the intervention group but were not able to report the rate in the control group.

The remaining studies were assessed for potential bias according to the US Preventive Services Task Force (USPSTF) quality rating criteria [17]. Review of bias assessments were completed independently by 2 reviewers (MA and WA). Any disagreements arising between the reviewers were resolved by discussion until a consensus was achieved.

### Data Extraction and Synthesis

Data were extracted regarding the study design, study procedure, intervention, population demographics, and number of patients having an exacerbation. Two reviewers (MA and WA) extracted data independently. Data from 3 studies were pooled using Review Manager version 5.3 (The Nordic Cochrane Centre, The Cochrane Collaboration, Copenhagen) [18]. A random effects model was used to pool results from the included studies and calculate a summary odds ratio to measure the independent effect of smartphone interventions on COPD exacerbations. We tested for variance across studies using the chi-square test and measured the degree of heterogeneity using the *I*^2^ statistic.

## Results

### Overview

**Figure 1:**
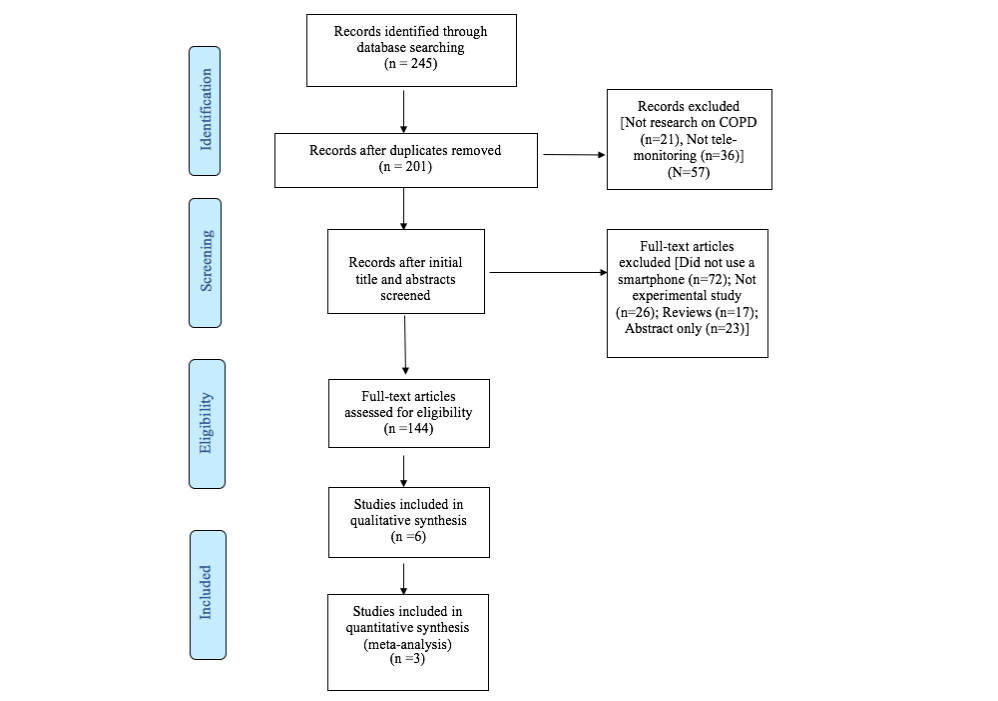
Preferred Reporting Items for Systematic Reviews and Meta-Analyses flow diagram of search results and study selection. COPD: chronic obstructive pulmonary disease.

The study selection process is outlined in Figure 1. The search process yielded 245 records, providing 201 citations after duplicates were removed. Of these, 6 studies met the eligibility criteria [19-24].

### Qualitative Analysis

Six studies were included in the qualitative analysis. Table 2 provides characteristics of the 6 included research studies [19-24]. All the articles were published after 2008. All of the studies were conducted on relatively small samples, less than 100 participants each. Some research studies specified the COPD severity stage according to the GOLD guidelines [21-23], whereas other studies included patients in all COPD stages [19,20,24]. Furthermore, patients were required to be free from COPD exacerbations for either at least 3 weeks [21] or one month [19,20,23,24] to be included in the research studies. Studies included older participants; the mean age was 70.5 years (SD 5.6). All studies had a large percentage of male participants (mean 74%).

Table 3 provides characteristics of the methodology used in the research studies [17-23]. The studies were conducted in various countries around the world. Five of the six included studies were randomized controlled trials [19-21,23,24], and one study used a quasi- experimental design [22]. Postintervention follow-up assessment for the included studies ranged between 4 months and 9 months. The smartphone in each study was primarily used to collect data about the daily symptoms of the patient. As a complement to the smartphone intervention, education about self-management and exercise training [19,22,24] was also used in some studies. Participants used the smart phone to report physical activity level [24], daily symptoms [19-24], and heart rate and oxygen saturation [21]. One study provided a Web portal to enable patients to treat exacerbations themselves [24]. All studies compared a smartphone intervention versus usual care as the control group, except one study. Tabak et al provided both the intervention and control groups with a smartphone, but only the intervention group received automated phone calls to remind the participants about the treatment regimen and to ensure that they had sufficient medications [24]. All studies provided participants with a smartphone but did not report other incentives to participate in the study.

The frequency of collecting data from participants was different between studies. Symptoms and objective measurements such as spirometry and pulse oximetry were collected on a daily basis. Alternatively, physical activity data were collected weekly. The investigators assessed collected data on a daily basis. When an exacerbation was detected, patients were contacted to confirm the exacerbation. One study used an automated feedback mechanism that advised to start medication in case of an exacerbation [23].

**Table 2:** Characteristics of studies using smartphone interventions with COPD patients.

First author, (year)	COPD^a^ stage	FEV_1_^b^, mean (SD), % predicted		Participant age (years), mean (SD),		Male sex, %		Sample size (analyzed)		No. of patients having an exacerbation	
		IG^c^	CG^d^	IG	CG	IG	CG	IG	CG	IG	CG
Tabak, (2014) [24]	All stages	48.7 (16.7)	56.4 (10.6)	65.2 (9.0)	67.9 (5.7)	57%	68%	15 (10)	15 (2)	33	N/R^e^
Pedone, (2013) [21]	II or III	52.5 (14.9)	55.4 (15.8)	74.1 (6.4)	75.4 (6.7)	72%	63%	50 (39)	49 (49)	9	15
Jehn, (2013) [23]	II-IV	50.2 (15)	52.6 (17.5)	64.1 (10.9)	69.1 (9.2)	81%	73%	32 (32)	30 (30)	7	22
Halpin, (2011) [20]	All stages	48 (4)	54 (3)	68.5 (1.5)	70.2 (1.6)	74%	73%	40 (39)	39 (38)	23	26
Nguyen, (2008) [19]	All stages	49.0 (16.8)	50.3 (17.6)	68.0 (8.3)	70.9 (8.6)	61%	55%	26 (20)	24 (19)	10	N/R
Liu, (2008) [22]	II or III	45.2(3.2)	46 (2.8)	71.4 (1.7)	72.8 (1.3)	100%	100%	30 (24)	30 (24)	2	10

^a^COPD: Chronic Obstructive Pulmonary Disease.

^b^FEV_1_: Forced Expiratory Volume in one second.

^c^IG: Intervention Group.

^d^CG: Control Group.

^e^N/R: not reported.

**Table 3:** Summary of the methodology in studies using smartphone interventions with COPD patients.

First author, (year)	Design (Follow-up) Country	Intervention (Frequency)	Control
Tabak, (2014) [24]	RCT^a^ (9 months) Netherlands	Short respiratory symptoms questionnaires, exercise program and self-management recommendations on the Web portal (Daily); Activity coach via an accelerometer and a smartphone (4days/week).	Usual care
Pedone, (2013) [21]	RCT (9 months) Italy	Heart rate, physical activity, near-body temperature, and galvanic skin response via wristband coupled with a smartphone (Every 3 hours); Oxygen saturation levels via a portable pulse oximeter (Every 3 hours). A physician contacted participants to provide feedback in case of abnormal readings (Daily).	Usual care
Jehn, (2013) [23]	RCT (9 months) Germany	COPD Assessment Test on the smartphone (Daily); Lung Function Tests via a portable spirometer (Daily). Six-minute walk test measured by accelerometer (Weekly). A study nurse contacted the participant to remind them about entering data (Daily).	Usual care
Halpin, (2011) [20]	RCT (4 months) United Kingdom	The Exacerbations of Chronic Pulmonary Disease Tool (EXACT) questionnaire on the smartphone (Daily); Automated phone calls to remind patients about the treatment regimen and ensure they have sufficient medication (Weekly).	EXACT questionnaire on the smartphone
Nguyen, (2008) [19]	RCT (6 months) United States	Exercise training program via smartphone (Daily); Short respiratory symptoms questionnaires on the smartphone (Daily). A study nurse contacted the participant to remind them about entering data and provide feedback (Daily).	Usual care
Liu, (2008) [22]	**NRCT**^b^ (9 months) Taiwan	Home-based endurance exercise training program via smartphone (Daily); Short respiratory symptoms questionnaires on the smartphone (Daily).	Usual care

^a^RCT: Randomized Controlled Trial.

^b^NRCT: Nonrandomized Controlled Trial.

### Quantitative Analysis

**Figure 2:**
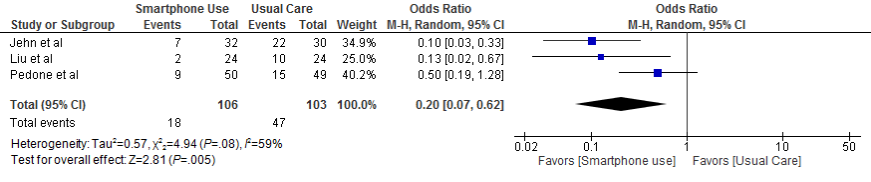
Effects of smartphone interventions on the number of patients having a COPD exacerbation.COPD: Chronic Obstructive Pulmonary Disease.

Three studies were included in the meta- analysis [21-23]. Two studies were excluded because they did not report the number of patients having an exacerbation in the control group [19,24], and another study provided a smartphone intervention to both the intervention and control groups [20]. The follow-up period for all 3 studies was 9 months. All 3 studies reported that participants receiving smartphone interventions experienced a reduction in COPD exacerbations [21-23]. Two studies used intention-to-treat analysis [21,23] and one study used per-protocol analysis [22]. The pooled odds ratio of patients having an exacerbation was 0.20 in the patients using a smartphone intervention (95% CI 0.07-0.62) compared with those receiving usual care. The meta-analysis of COPD exacerbations indicates a reduction of 80% for smartphone interventions compared with usual care. There was moderate heterogeneity across the studies that were included in the meta-analysis (χ^2^_2_=4.9, *P*=.08, *I*^2^=59%) [25]. The results are outlined in Figure 2.

### Risk of Bias

A summary of the assessment of potential bias of studies selected for inclusion, using USPSTF Quality Rating Criteria, can be found in Table 4. The overall assessment of the studies that were included in the meta-analysis was Good [23] and Fair [21,22]. It was not possible to assess for publication bias via funnel plot asymmetry due to the low number of studies included in the meta-analysis [26].

**Table 4:** Assessment of potential bias of studies selected for inclusion using USPSTF Quality Rating Criteria [16].

Study	Assembly of comparable groups	Maintenance of comparable groups	No important differential loss to follow-up or overall high loss to follow-up	Measurements: equal, reliable, valid (includes masking of outcome assessment)	Clear definition of interventions	All-important outcome considered	Analysis: adjustment for potential confounders	Overall assessed quality
Nguyen (2008) [19]	Good	Fair	Good	Fair	Good	Good	Poor	Fair
Halpin (2011) [20]	Good	Good	Fair	Fair	Poor	Fair	Fair	Fair
Pedone (2013) [21]	Fair	Good	Good	Fair	Good	Fair	Good	Fair
Liu (2008) [22]	Fair	Good	Fair	Fair	Good	Good	Fair	Fair
Jehn et (2013) [23]	Fair	Good	Good	Fair	Good	Good	Good	Good
Tabak (2014) [24]	Poor	Fair	Poor	Fair	Good	Good	Fair	Poor

## Discussion

### Principal Results

The existing literature indicated that there is a potential for smartphone interventions in reducing the frequency of COPD exacerbations. Although most COPD patients were older than 65 years, they were able to use smartphones to monitor their symptoms. Rates of COPD exacerbations among participants receiving a smartphone intervention during the trials proved to be less compared with the participants not receiving a smartphone intervention. The main objective for using a smartphone is early identification of COPD exacerbations. Early identification allows the patient and health care team to intervene successfully, thus improving the management of COPD and reducing COPD exacerbations. As stated previously, Najafzadeh et al indicate that any intervention that reduces the number of exacerbations has a substantial impact on morbidity and costs of COPD [6].

Our finding that smartphones could be useful in reducing COPD exacerbations replicates the findings of 3 cohort studies. Jarad and Sund coupled a smartphone with a portable spirometer and indicated that it reduced the number of hospitalizations for COPD exacerbations [27]. Johnston et al showed that smartphone-based collection of COPD symptom diaries allows patients to identify exacerbation symptoms early on in the exacerbation allowing for early intervention [14]. Furthermore, Ding et al conducted a cohort study of a mobile phone–based home monitoring system and demonstrated the potential of smartphones in early identification of COPD exacerbations [28]. Thakkar et al conducted a systematic review and stated that mobile phone text messaging approximately doubles the odds of medication adherence in patients with chronic diseases [29]. Smartphones can incorporate text-messaging interventions in addition to various interventions that include, but are not limited to, surveys, reminders, and the ability to be paired with medical devices.

### Risk of Bias

Although the included studies reported promising results, there was moderate heterogeneity (*I*^2^=59%) across studies that were included in the meta-analysis. Liu et al [22] did not randomize patients to the intervention while the other 2 studies conducted randomized controlled trials [21,23]. The studies also varied in location, COPD severity, smartphone intervention, frequency of data collection from the participants, and the feedback strategy.

In many studies, the smartphone intervention was combined with different variations of symptoms diaries, physiological monitoring, and educational elements directed at patients. Patients used the smartphone to report daily symptoms [22,23] or deliver a home-based exercise training program [21]. In addition, investigators coupled the smartphone with various medical devices to measure physical activity levels [21,23], heart rate and oxygen saturation [21], and pulmonary function tests [22]. Each intervention, patient education or use of medical devices, could itself account for the differences between groups. Therefore, researchers should be cautious when interpreting the synergistic effect from the combination of these interventions.

The frequency of data collection from participants and feedback strategy also differed between the studies. Liu et al collected data from participants every day [22]. The data was reviewed weekly and feedback was given to participants during their three-month follow up visits. Jehn et al collected data from participants every day and physicians reviewed the data daily; however, the feedback strategy to patients was unclear [23]. Pedone et al collected data more frequently than other studies due to the use of the wristband and portable pulse oximeter [21]. Unusual data were flagged and physicians assessed the data on a daily basis. Then, physicians contacted the participants to assess for a COPD exacerbation and suggest an intervention.

Only 2 studies reported on metrics related to user experience [19,24]. Nugyen et al conducted semistructured interviews with participants at the end of the study [19]. Participants were asked to provide feedback on what aspects of the program were most or least helpful for managing their dyspnea and how the program could have been done differently to support self-management. On the other hand, Tabak et al used the Client Satisfaction Questionnaire to measure user satisfaction [24]. Unfortunately, we were unable to combine the usability results due to the differences in the methods used to measure user experience.

The frequency of data collection from the participant was also dependent on the type of data being collected. Symptoms were collected daily while exercise progress was assessed weekly. Collecting data from participants frequently could yield more accurate data; nevertheless, it must not compromise the participant’s adherence to the intervention. There are many factors that could have caused the reduction in COPD exacerbation. Early detection of symptoms and timely treatment could be possible by the use of smartphones or due to phone contact by the research team. Currently, we are uncertain whether the reduction in the number of patients having an exacerbation is caused by the smartphone intervention or merely due to bias among the studies. Additional investigations are required before large-scale implementation of smartphone interventions.

### Limitations

Aside from the methodological heterogeneity among studies, there are several limitations with this systematic review. There are a limited number of studies using smartphones in the management of COPD exacerbations, each with relatively small samples, less than 100 participants each. A comprehensive search strategy was used, but studies utilizing smartphones in the management of COPD exacerbations that are still in progress or provided only an abstract were excluded. All investigators provided a smartphone to participants. This could have caused highly motivated participants who are familiar with smartphones to contribute data. Another limitation is that studies did not clearly define exacerbations (recognized and unrecognized) and how to identify it (eg, drug use, reported symptoms, and emergency admission). Tabak used a self-management Web portal to measure exacerbations, which could have yielded many false positive results. The review favored smartphone interventions across all studies, thus overall findings do indicate that smartphone interventions may reduce the number of patients having COPD exacerbations across a wide variety of contexts.

### Implications for Future Research

Implementing a mixed methods research design to investigate the validity and clinical utility of smartphone interventions could help to understand why a particular component is successful and how patients will use smartphone interventions for a long-term. There is limited research regarding smartphone interventions among COPD patients. Although the studies in this review have a small sample size and a relatively short follow-up period, current literature supports the potential of smartphones in reducing COPD exacerbations. There is a need for more studies evaluating smartphone interventions, including studies using smartphones as the main intervention. This will assist in determining whether smartphones can be effective in the management of COPD. Investigators should include participants with different stages of COPD severity and age spans to minimize the risk of bias and enhance the generalizability of the study results.

### Conclusion

Although the current literature on the role of smartphones in reducing COPD exacerbations is limited, our results suggest that smartphone interventions may reduce COPD exacerbations. Nevertheless, using smartphones require synergistic strategies to achieve the desired outcome. The results should be interpreted with caution due to the heterogeneity among the studies, risk of small study bias, and limitations in study quality. Researchers should focus on conducting rigorous randomized controlled trial (RCT) studies with adequately powered sample sizes to determine the validity and clinical utility of smartphone interventions in the management of COPD.

## Abbreviations

CG: Control Group
COPD: Chronic Obstructive Pulmonary Disease
FEV1: Forced Expiratory Volume in one second
GOLD: Global initiative for chronic Obstructive Lung Disease
IG: Intervention Group
N/R: Not Reported
NRCT: Nonrandomized Controlled Trial
PRISMA: Preferred Reporting Items for Systematic Reviews and Meta-Analyses
RCT: randomized controlled trial
USPSTF: US Preventive Services Task Force
